# Pulp Treatment

**Published:** 1882-09

**Authors:** J. B. Hodgkin


### EDITORIAL, ETC.

*Pulp Treatment.*—Dr. Bödecker, in the New York Odontological Society meeting in April last, states that it is practicable to destroy the periphral portion of a tooth pulp, with arsenious acid, and that action of this agent, if continued any twenty-four hours, is periphral only. If continued longer it devitalizes the entire pulp. He states that it is possible in this way destroy a pulp on the surface only, and that amputation of the divitalized portion may then be perfomed, with a saving of the remaining portion of the organ. In the amputation he goes below the "line of demarkation," and into the healty tissue beyond. The language is "a great deal below the line of demarkation." Amputation of a part of the pulp may be performed thus with a sharp bur, or excised in any convenient manner. Dr. B. holds that the remaining portion of the pulp will perform its functions satisfactorily. He quotes from Witzel, whom he represents as saying that he has so far performed six hundred amputations of the pulp without a single failure.

Certainly if one can get parts of pulps to live it will be a great blessing, especially in molar teeth,, where the filling of the roots is impracticable. The doctrine is not new, but has been so disputed that we remember hearing celebrated men, among others, college professors, saying that they had never seen a case of successful nerve capping. This, of course is not true of all observers, but that many failures occur is patent.

If this has been due to leaving diseased portions of the pulp in position, and if amputation with the aid of arsenic is practicable, and if the pulp after such amputation and deprivation of a part of its substance will still perform its functions, we may take courage.

J. B. HODGKIN.

